# Diagnostic Utility of BAP1, EZH2 and Survivin in Differentiating Pleural Epithelioid Mesothelioma and Reactive Mesothelial Hyperplasia: Immunohistochemical Study

**DOI:** 10.3389/pore.2021.600073

**Published:** 2021-04-09

**Authors:** Sarah Adel Hakim, Hoda Hassan Abou Gabal

**Affiliations:** Department of Pathology, Faculty of Medicine, Ain Shams University, Cairo, Egypt

**Keywords:** mesothelioma, reactive mesothelial hyperplasia, BAP1, EZH2, survivin, immunohistochemistry

## Abstract

**Background:** Epithelioid mesothelioma (EM) is the commonest subtype of malignant pleural mesothelioma. Its histopathological discrimination from reactive mesothelial hyperplasia (RMH) could be challenging. Thus, an immunohistochemical panel is mandatory for better discrimination. BAP1 is a newly identified diagnostic marker whose loss is specific to malignant mesothelioma. EZH2 overexpression is reported in different cancers, but its relation to BAP1 in malignant mesothelioma has not been fully understood. Survivin expression is said to be significantly higher in EM than in non-neoplastic pleural tissue, but its diagnostic utility as an immunohistochemical marker has not been thoroughly investigated in this field. To the best of our knowledge, no previous studies have been conducted to assess the diagnostic accuracy of the combined use of these three nuclear markers (BAP1, EZH2 and Survivin) in discriminating pleural EM from RMH.

**Methods:** This retrospective study includes two groups: 81 cases of pleural EM and 67 cases of RMH, retrieved from the archives of Pathology Department of Ain Shams University Hospitals and Ain-Shams University Specialized Hospital during the period from January 2016 to December 2019. An immunohistochemical study was performed using BAP1, EZH2 and Survivin antibodies.

**Results:** There were highly statistically significant relations between study groups as regards the studied markers (*p* = 0.001 for each). The specificity was 100% for all combinations of immunohistochemical markers. Sensitivity of any combination of the immunohistochemical markers used in this study was found to be higher than the sensitivity of any of these markers used individually. The combination of all three markers showed the highest diagnostic accuracy (95.9%) and the highest sensitivity (92.6%). However, the combination of Survivin and EZH2 yielded the same diagnostic accuracy and sensitivity.

**Conclusion:** Adding EZH2, Survivin and BAP1 to the diagnostic IHC panel for differentiating pleural EM and RMH could enhance diagnostic sensitivity. Moreover, Survivin is a potentially promising marker in this context, especially when combined with EZH2.

## Introduction

Pleural malignant mesothelioma poses a diagnostic challenge that may cause late diagnosis at an advanced stage. Being an extremely aggressive cancer, it requires a highly sensitive and specific panel of immunohistochemical markers capable of early and definitive diagnosis [1].

BRCA1-associated protein 1 (BAP1) is a newly identified diagnostic marker whose loss is specific to malignant mesothelioma [2, 3]. BAP1 expression was recorded in up to 77% of epithelioid mesothelioma (EM). BAP1 loss is caused by mutations, deletions or epigenetic silencing of BAP1 in both familial and sporadic malignant mesothelioma. It encodes ubiquitin C-terminal hydrolase that may have a role in keeping the appropriate ubiquitination status of target histones. Nonetheless, the definite role of BAP1 loss in malignant transformation of mesothelial cells is still ambiguous to a great extent [4].

It was demonstrated that BAP1 loss promoted cell proliferation *in vitro* through up-regulation of enhancer of zeste 2 polycomb repressive complex two subunit (EZH2) [5]. EZH2 encodes a histone-lysine N-methyltransferase that functions as a transcriptional repressor. EZH2 overexpression was reported in different cancers, such as prostatic cancer, breast cancer, and uterine cancer, and had worse prognosis in some cancers [6]. In malignant mesothelioma, it was demonstrated that EZH2 mRNA expression was elevated, yet EZH2 IHC expression and its association with BAP1 in malignant mesothelioma have not been fully understood.

Although the primary function of EZH2 is gene silencing through the methylation of H3K27, most evidence shows that EZH2 functions independently of H3K27me3 in various cancers; for instance, in some cancers EZH2 was shown to interact with β-catenin and promote its nuclear accumulation and activation [7], to form the β-catenin/T-cell factor (TCF) transcriptional activator, which up-regulates a number of target genes such as survivin, c-Myc and VEGF [8]. It was reported that the level of mRNA expression of baculoviral IAP repeat-containing 5 (BIRC5; Survivin) was significantly higher in EM than in non-neoplastic pleural tissue [9], although the utility of Survivin IHC in differentiating benign and malignant mesothelial proliferation has not yet been thoroughly investigated.

To the best of our knowledge, no previous studies were conducted to assess the diagnostic accuracy of the combined use of BAP1, EZH2 and Survivin in discriminating pleural epithelioid mesothelioma from reactive mesothelial hyperplasia. Thus, the current study aims at assessing their diagnostic utility, combined together, in different pairs, and individually while comparing the diagnostic accuracy in each condition.

## Material and Methods

### Tissue and Patient Data

The current study was conducted on two groups: 81 cases of pleural epithelioid mesothelioma (EM) and 67 cases with reactive mesothelial hyperplasia (RMH). Cases of both groups were obtained from the archives of the Pathology Lab. of Ain-Shams University Specialized Hospital and Ain-Shams University Hospitals. The study cases were diagnosed during the period from January 2016 to December 2019; and cases of both groups were obtained via thoracoscopic pleural biopsy. The histopathology reports were reviewed to determine age and sex of patients. Haematoxylin and Eosin stained slides were examined to re-evaluate and verify the histopathologic diagnosis. In addition, samples from the EM group were evaluated to determine growth pattern, degree of nuclear atypia, presence of necrosis and tumor grading using two-tier system [10]. International Mesothelioma Interest Group (IMIG) staging [11] was available for the EM cases included in the study. Only primary samples of EM patients who did not receive prior neoadjuvant therapy as well as samples with enough tissue and information on all covariates were selected in the analysis.

### Ethics Statement

All patients who participated in this study signed a written informed consent before thoracoscopic pleural biopsy. The study was approved by the Research Ethical Committee at Faculty of Medicine, Ain Shams University.

### Immunohistochemical Staining

Four micrometer sections of formalin-fixed and paraffin-embedded samples of pleural EM and RMH were prepared. Immunohistochemical staining was performed using primary antibodies: rabbit polyclonal anti-Survivin (Clone: AF886; R&D systems, MN, United States; dilution of 1:200); mouse monoclonal anti-BAP1 (Clone: C-4; Santa Cruz Biotechnology, Santa Cruz, CA, United States; 1:100 dilution) and mouse monoclonal anti-EZH2 (11) (Clone: 415M-18; Cell Marque, Sigma-Aldrich Co.,CA, United States; 1:100 dilution). Avidin-Biotin immunoperoxidase complex technique was used according to the study of Hsu et al. [12], by applying the super sensitive detection kit (Biogenex, CA, United States). The prepared tissue sections were fixed on poly-L- lysine coated slides overnight at 37°C. They were deparaffinized and rehydrated through graded alcohol series. Then the sections were heated in a microwave oven in 10 mM citrate buffer (pH 6.0) for 20 min. After the blocking of endogenous peroxidase and incubation in Protein Block Serum-Free Solution (Dako Cytomation, Glostrup, Denmark) for 20 min, the sections were incubated overnight at 4°C with primary antibodies. Biotinylated anti-mouse immunoglobulin and streptavidin conjugated to horseradish peroxidase were then added. Finally, 3,3′-diaminobenzidine, as the substrate or chromogen, was used to form an insoluble brown product. Finally, the sections were counterstained with hematoxylin and mounted. With each run, sections of human pancreas tissue, breast invasive duct carcinoma and non-Hodgkin lymphoma (NHL) were used as a positive control for BAP-1, EZH2 and Survivin respectively [12–14]. Negative control sections were incubated with normal mouse serum instead of the three primary antibodies.

### Interpretation of Immunohistochemical Staining

Immunohistochemical analysis of BAP1, EZH2 and Survivin was blindly performed by the two pathologists (the authors) without any prior knowledge of the clinicopathological data. Any discrepancies were resolved by consensus using a multi-head microscope.

Nuclear staining of BAP1, EZH2 and Survivin in EM or RMH cells with similar or higher intensity, as positive control tissue, was regarded as positive staining. For BAP1, negative staining was defined as completely absent nuclear staining in the target cells [16].

Immunoreactivity for EZH2 was divided into two groups; *no/low expression* (proportion of cells <50%; which included EZH2 null with a cut-off of <10% positive tumor cells and low EZH2 with a cut-off of 10–49% positive tumor cells), or *high expression* (proportion of cells of ≥50%). This was done for easier statistical analysis and guided by previous papers [4, 15].

Immunoreactivity for Survivin was evaluated using a labeling index (% of positive cells) in the “hot spot” exhibiting the highest number of positive cells compared to the rest of the lesion. At least 100 EM or RMH cells in high power fields (×400) were evaluated. Independent counting of labeling indices of Survivin was performed by the two pathologists (the authors), then the mean of the two evaluations was calculated [13]. The cut-off value for the Survivin IHC evaluation was set at 5% guided by a previous study [16]; hence, immunoreactivity of Survivin was classified as negative (positivity of less than 5% of mesothelioma cells or non-neoplastic mesothelial cells), or positive (positivity of ≥5% of the target cells).

Some background inflammatory cells/stromal cells showed positivity for these nuclear markers and served as internal positive control. Careful observation under high power magnification was performed in all cases for better discrimination of tumor cells. Any interobserver variance was decided by consensus using a multi-head microscope.

### Statistical Analysis

Continuous variables are expressed as mean and Standard Deviation. Categorical variables are expressed as frequencies and percent points. Student *t* test was used to assess the statistical significance of the difference between two study group mean. Chi square and Fisher’s exact test were used to examine the relationship between Categorical variables. A significance level of *p* < 0.05 was used in all tests. All statistical procedures were carried out using SPSS version 15 for Windows (SPSS Inc., Chicago, IL, United States).

## Results

### Clinicopathological Characteristics

The study includes 81 cases of pleural epithelioid mesothelioma (EM) and 67 cases of reactive mesothelial (RMH) hyperplasia. Sixty-four out of the 81 EM cases are males and 17 are females; and out of the 67 RMH, 59 cases are males, while only eight are females. The mean age of EM is 67.9 years (SD ± 3.4), while the mean age of RMH is 47.4 years (SD ± 2.3).

There is a highly significant difference among the study groups as regards age where EM was observed among older age groups. No significant difference was observed with respect to gender as males are predominant in both groups (Table 1).

**TABLE 1:** Comparison between the two study groups as regards personal data.

		Group				*p*	Sig
		Pleural mesothelioma		RMH			
		Mean	±SD	Mean	±SD		
Age		67.9	3.4	47.4	2.3	0.001^a^	HS
		**N**	**%**	**N**	**%**		
Gender	Male (n %)	64	79.0%	59	88.1%	0.144^b^	NS
	Female (n %)	17	21.0%	8	11.9%		

^a^ Student t test.

^b^ Chi-Square Tests.

Concerning the EM group, data distribution of clinicopathological parameters are summarized in Table 2.

**TABLE 2:** Clinicopathologic parameters of EM cases.

		Mean	±SD	Minimum	Maximum
Age		67.88	3.44	60.00	74.00
		**N**	**%**		
Gender	Male	65	80.2%		
	Female	16	19.8%		
Growth pattern	Solid	46	56.8%		
	Tubulopapillary	29	35.8%		
	Trabecular	6	7.4%		
Two-tier grade	Low	67	82.7%		
	High	14	17.3%		
Presence of necrosis	Absent	57	70.4%		
	Present	24	29.6%		
Degree of nuclear atypia	Mild	8	9.9%		
	Moderate	59	72.8%		
	Severe	14	17.3%		
IMIG stage	Early stages (I/II)	42	51.9%		
	Late stages (III/IV)	39	48.1%		

### Immunohistochemical Results

**FIGURE 1:**
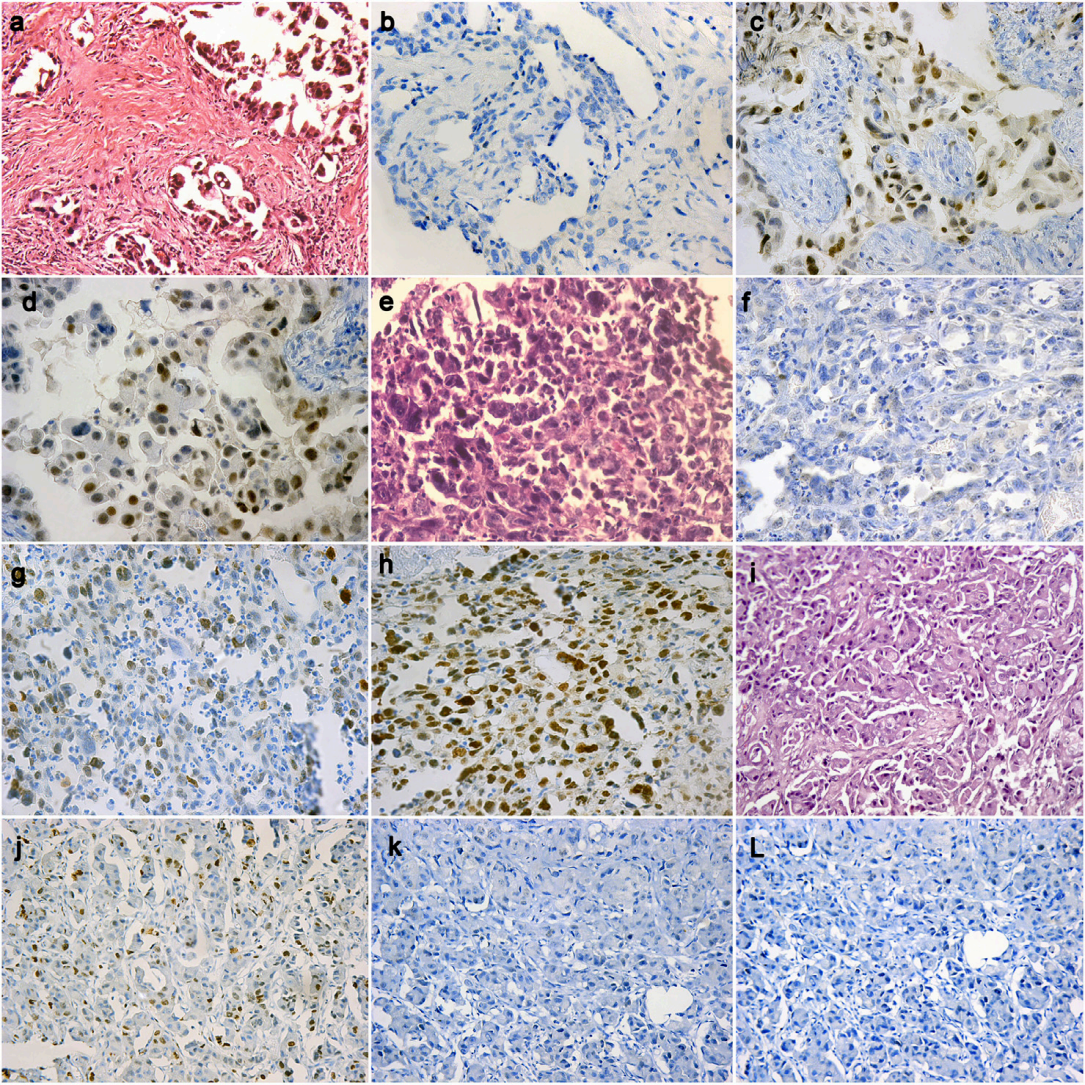
A case of epithelioid mesothelioma (H&E ×20) **(A)**, BAP1 loss in tumor cells (IHC ×40) **(B)**, High EZH2 nuclear expression in tumor cells (IHC ×40) **(C)**, Positive Survivin expression in tumor cells (IHC ×40) **(D)**; A case of epithelioid mesothelioma (H&E ×40) **(E)**, BAP1 loss in tumor cells (IHC ×20) **(F)**, Low EZH2 nuclear expression in tumor cells (IHC ×20) **(G)**, Positive Survivin expression in tumor cells (IHC ×20) **(H)**; A case of epithelioid mesothelioma (H&E ×20) **(I)**, BAP1 retained in tumor cells (IHC ×20) **(J)**, Negative EZH2 nuclear expression in tumor cells (IHC ×20) **(K)**, Negative Survivin expression in tumor cells (IHC ×20) **(L)**.

**FIGURE 2:**
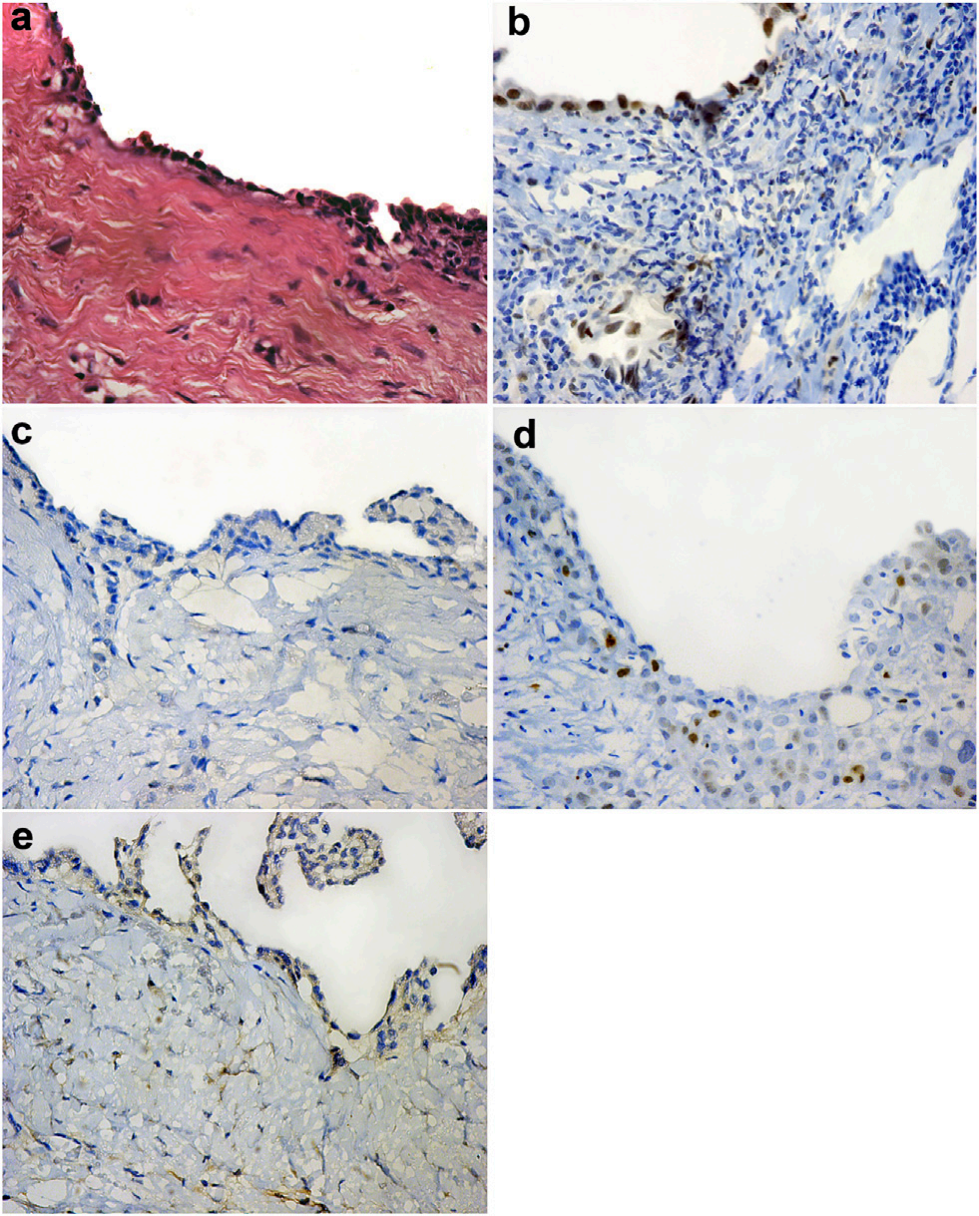
Reactive mesothelial hyperplasia (H&E ×40) **(A)**, Positive BAP-1 nuclear expression in reactive mesothelial cells (IHC ×40) **(B)**, Negative EZH2 nuclear expression in reactive mesothelial cells (IHC ×40) **(C)**, Low EZH2 nuclear expression in reactive mesothelial cells (IHC ×40) **(D)**, Negative Survivin nuclear expression in reactive mesothelial cells (IHC ×40) **(E)**.

Forty-nine (60.5%) out of the 81 cases of pleural EM reveal BAP1 homogenous expression loss pattern (Figures 1B,F) while the rest of the cases show retained BAP1 nuclear expression (Figure 1J) (Table 3). On the other hand, all 67 RMH cases show retained BAP1 nuclear expression (Figure 2B).

**TABLE 3:** Comparison between the two study groups as regard studied markers individually and in combinations.

		Group				*p*	Sig
		Pleural mesothelioma		RMH			
		N	%	N	%		
Survivin IHC expression	Positive	55	67.9	0	0.0	0.001	HS
	Negative	26	32.1	67	100.0		
BAP1 IHC expression	Loss	49	60.5	0	0.0	0.001	HS
	Retained	32	39.5	67	100.0		
EZH2 IHC expression	High	36	44.4	0	0.0	0.001	HS
	No/Low	45	55.6	67	100.0		
EZH2 IHC expression	High	36	44.4	0	0.0	0.001^a^	HS
	Low	32	39.5	7	10.4		
	Null	13	16.1	60	89.6		
Survivin-positive and/or BAP1-loss	Positive	61	75.3	0	0.0	0.001^a^	HS
	Negative	20	24.7	67	100.0		
Survivin-positive and/or EZH2-High expression	Positive	75	92.6	0	0.0	0.001^a^	HS
	Negative	6	7.4	67	100.0		
BAP1-loss and/or EZH2-High expression	Positive	69	85.2	0	0.0	0.001^a^	HS
	Negative	12	14.8	67	100.0		
Survivin-positive and/or BAP1-loss and/or EZH2-High expression	Positive	75	92.6	0	0.0	0.001^a^	HS
	Negative	6	7.4	67	100.0		

^a^ Chi-Square Tests.

Thirty-six (44.4%) out of 81cases of pleural EM show high EZH2 nuclear expression (Figure 1C). However, none of the 67 cases of RMH show high EZH2 nuclear expression (Figure 2C,D), (Table 3). No EZH2 expression could be detected in the adjacent normal mesothelial cells.

Fifty-five (67.9%) out of 81 cases of pleural EM show positive Survivin nuclear expression (Figure 1D,H). However, all 67 cases of RMH show negative Survivin nuclear expression (Figure 2E), (Table 3).

There is a highly significant difference between study groups and the studied markers; individually and in different combinations as shown in Table 3.

### Sensitivity and Specificity of the Immunohistochemical Markers

The sensitivity and specificity of each marker for discriminating pleural EM from RMH cases was summarized in Table 4. All of Survivin positivity, BAP1 loss, and high expression of EZH2 expressed in cases of EM had 100% specificity, and their sensitivities were 67.9, 60.5, and 44.4%, respectively.

**TABLE 4:** Diagnostic utility of studied markers in malignant mesothelioma; individually and in combinations.

	Sensitivity (%)	Specificity (%)	PPV (%)	NPV (%)	Accuracy (%)
Survivin positive	67.9	100.0	100.0	72.0	82.4
BAP1-loss	60.5	100.0	100.0	67.6 5	78.3
EZH2 high expression	44.4	100.0	100.0	59.8	69.5
Survivin-positive and/or BAP1-loss	75.30	100	100	77	86.5
**Survivin-positive and/or EZH2-High expression**	**92.60**	**100**	**100**	**91.7**	**95.9**
BAP1-loss and/or EZH2-High expression	85.20	100	100	84.8	91.9
**Survivin-positive and/or BAP1-loss and/or EZH2-High expression**	**92.60**	**100**	**100**	**91.7**	**95.9**

Bold values indicate combinations with the highest diagnostic accuracy.

The specificity was 100% for all combinations of immunohistochemical markers. Sensitivity of any combination of the immunohistochemical markers used in this study is higher than the sensitivity of any of these markers alone. Although the combination of all three markers showed the highest diagnostic accuracy (95.9%) and the highest sensitivity (92.6%), still the combination of only Survivin and EZH2 yielded the same diagnostic accuracy and sensitivity, which showed that adding BAP1 to the combination did not improve the diagnostic accuracy or sensitivity (Table 4).

### Association Between the Immunohistochemical Markers

**FIGURE 3:**
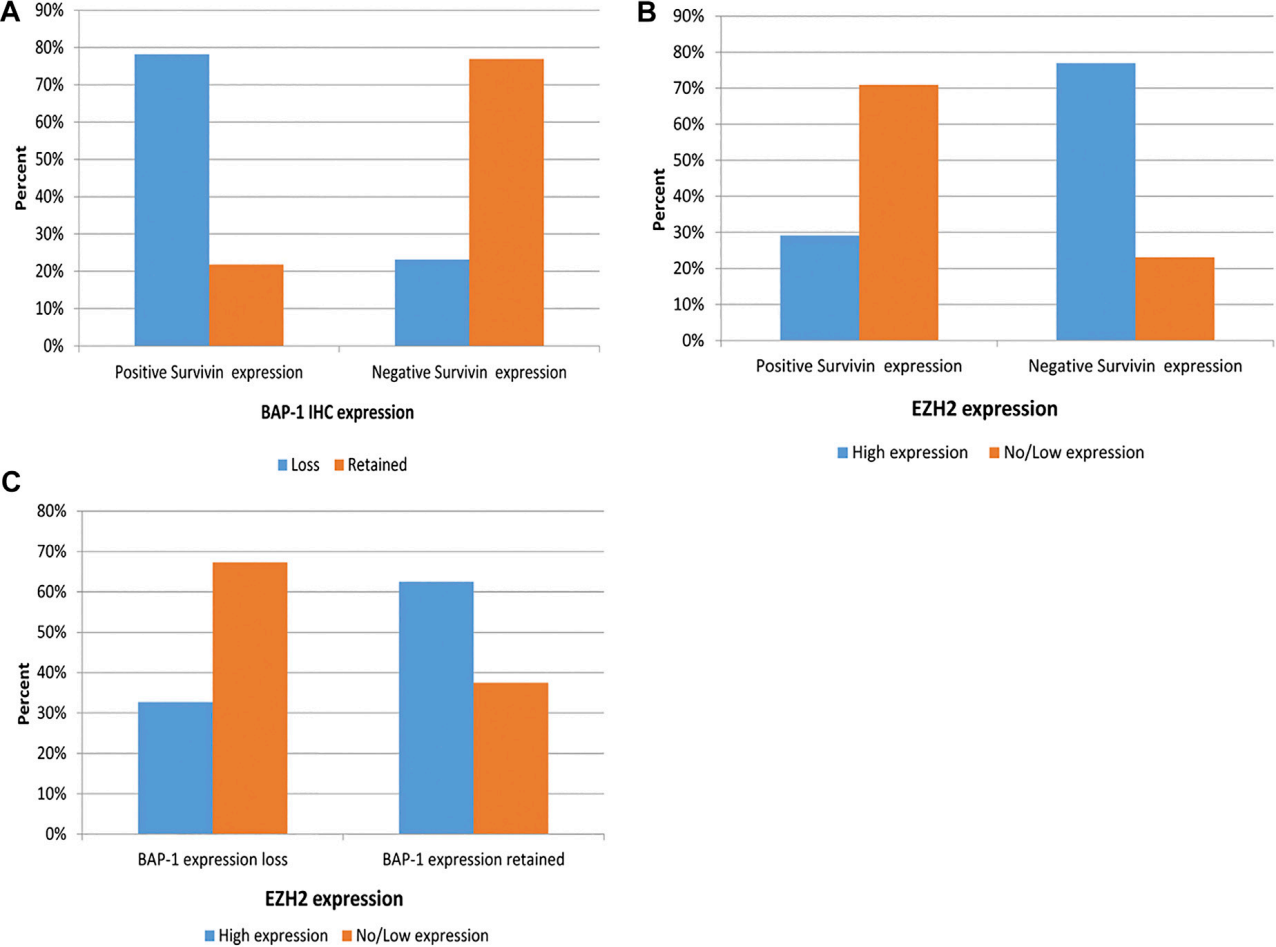
Association between Survivin IHC and BAP1 IHC expression among EM cases **(A)**, Association between Survivin IHC and EZH2 IHC expression among EM cases **(B)**, Association between BAP1 IHC expression and EZH2 IHC expression among EM **(C)**.

There was a highly statistically significant association between positive Survivin IHC expression and BAP1 loss among mesothelioma cases, as 78% of positive Survivin cases had BAP1 loss compared to 23.1% only of negative Survivin IHC expression cases that manifested BAP1 loss IHC expression (Figure 3A). Also, a high statistically significant association between EZH2 and Survivin (*p* = 0.001) is detected, where 70.9% of positive Survivin malignant mesothelioma cases are associated with low EZH2 expression and high EZH2 expression in malignant mesothelioma is associated with negative Survivin expression in 76.9% of cases (Figure 3B). There was a statistically significant association between BAP1 and EZH2 expression (*p* = 0.008), where 67.3% of cases of BAP1 loss showed low EZH2 and 62.5% of malignant mesothelioma cases with retained BAP1 showed high EZH2 expression (Figure 3C).

### Relationship Between the Studied Markers and Clinicopathological Parameters in EM Group

Comparing IHC expression of the three studied markers to clinicopathological parameters of EM (growth pattern, two-tier system of grading, presence of necrosis, degree of nuclear atypia, IMIG staging) reveals no statistically significant relationships (Tables 5–7).

**TABLE 5:** Correlation between BAP1 IHC expression and clinicopathological parameters of EM group.

		BAP1 IHC expression				*p*	Sig
		Negative		Positive			
		Mean	±SD	Mean	±SD		
Age		67.98	3.51	67.72	3.39	0.741^a^	NS
		N	%	N	%		
Gender	Male	40	81.6%	25	78.1%	0.698^b^	NS
	Female	9	18.4%	7	21.9%		
Growth pattern	Solid	28	57.1%	18	56.3%	0.880^c^	NS
	Tubulopapillary	18	36.7%	11	34.4%		
	Trabecular	3	6.1%	3	9.4%		
Two-tier grade	Low	42	85.7%	25	78.1%	0.377^b^	NS
	High	7	14.3%	7	21.9%		
Presence of necrosis	Absent	34	69.4%	23	71.9%	0.811^b^	NS
	Present	15	30.6%	9	28.1%		
Degree of nuclear atypia	Mild	6	12.2%	2	6.3%	0.525^c^	NS
	Moderate	36	73.5%	23	71.9%		
	Severe	7	14.3%	7	21.9%		
IMIG stage	Early stages (I/II)	28	57.1%	14	43.8%	0.238^b^	NS
	Advanced stages (III/IV)	21	42.9%	18	56.3%		

^a^ Student t test.

^b^ Chi-Square Tests.

^c^ Fisher’s exact test.

**TABLE 6:** Correlation between EZH2 IHC expression and clinicopathological parameters of EM group.

		EZH2 IHC expression				*p*	Sig
		No/Low		High			
		Mean	±SD	Mean	±SD		
Age		68.18	3.08	67.50	3.86	0.382^a^	NS
		N	%	N	%		
Gender	Male	35	77.8%	30	83.3%	0.533^b^	NS
	Female	10	22.2%	6	16.7%		
Growth pattern	Solid	26	57.8%	20	55.6%	1.0^c^	NS
	Tubulopapillary	16	35.6%	13	36.1%		
	Trabecular	3	6.7%	3	8.3%		
Two-tier grade	Low	40	88.9%	27	75.0%	0.100^b^	NS
	High	5	11.1%	9	25.0%		
Presence of necrosis	Absent	35	77.8%	22	61.1%	0.103^b^	NS
	Present	10	22.2%	14	38.9%		
Degree of nuclear atypia	Mild	7	15.6%	1	2.8%	0.070^c^	NS
	Moderate	33	73.3%	26	72.2%		
	Severe	5	11.1%	9	25.0%		
IMIG stage	Early stages (I/II)	30	66.7%	12	33.3%	0.003^b^	HS
	Advanced stages (III/IV)	15	33.3%	24	66.7%		

^a^ Student t test.

^b^ Chi-Square Tests.

^c^ Fisher’s exact test.

**TABLE 7:** Correlation between Survivin IHC expression and clinicopathological parameters of EM group.

		Survivin IHC expression				*p*	Sig
		Negative		Positive			
		Mean	±SD	Mean	±SD		
Age		68.65	3.52	67.51	3.38	0.164^a^	NS
		N	%	N	%		
Gender	Male	21	80.8%	44	80.0%	0.935^b^	NS
	Female	5	19.2%	11	20.0%		
Growth pattern	Solid	14	53.8%	32	58.2%	0.880^c^	NS
	Tubulopapillary	9	34.6%	20	36.4%		
	Trabecular	3	11.5%	3	5.5%		
Two-tier grade	Low	21	80.8%	46	83.6%	0.750^b^	NS
	High	5	19.2%	9	16.4%		
Presence of necrosis	Absent	18	69.2%	39	70.9%	0.877	NS
	Present	8	30.8%	16	29.1%		
Degree of nuclear atypia	Mild	2	7.7%	6	10.9%	0.525	NS
	Moderate	19	73.1%	40	72.7%		
	Severe	5	19.2%	9	16.4%		
IMIG stage	Early stages (I/II)	15	57.7%	27	49.1%	0.469	NS
	Advanced stages (III/IV)	11	42.3%	28	50.9%		

^a^ Student t test.

^b^ Chi-Square tests.

^c^ Fisher’s exact test.

## Discussion

Accurate histopathological differentiation between malignant pleural mesothelioma (MPM) and reactive mesothelial hyperplasia (RMH) is extremely crucial [16]. The commonest subtype of MPM is epithelioid mesothelioma (EM), constituting about 50–70% of all cases of MPM [17]. EM shows variable degrees of architectural complexity and cytological atypia; therefore, cases of EM are not always easily distinguished from cases of RMH, especially in the instances where tumor cells have bland nuclei proliferating in tubulo-trabecular pattern with minimal stromal invasion. On the other hand, RMH could show severe proliferation of mesothelial cells with worrisome nuclear features. It usually shows no evidence of stromal invasion, yet care needs to be taken in assessing this as mesothelial entrapment may occur especially in inflammatory processes [4]. Therefore, to obtain a better diagnostic panel for EM, this study evaluated the diagnostic utilities of BAP1, EZH2 and Survivin in distinguishing EM from RMH.

BAP1 is a relatively new marker for the diagnosis of malignant mesothelioma and its utility has been demonstrated in several studies. The frequency of BAP1 loss demonstrated in EM cases in the present study was 60.5%, whereas none of the cases of RMH showed BAP1 loss. This was similar to what was previously reported by Shinzaki-Ushiku et al., who demonstrated BAP1 loss in 61% of their EM cases [4]. However, the percentage is somewhat lower than what was reported by Kushitani et al., whose frequency of BAP1 loss was 66.2% in EM; though their results agreed with those of the present study with respect to RMH cases, which also showed no BAP1 loss in any of them [18–20].

Concerning the pattern of staining of BAP1, in the current study, all EM cases had either a uniform positive nuclear staining pattern or completely negative nuclear staining for BAP1. This was similar to the pattern described by Kushitani et al. [16], but was different from that of Hida et al. [18] who demonstrated focal heterogenous BAP1 staining pattern in their mesothelioma cases. This may be attributed to the presence of inflammatory cells infiltrating into the mesothelioma or stromal cells that necessitate careful observation under high power magnification. Other reasons, such as differences in staining techniques and improper processing of the tumor may also contribute to the apparent differences among the studies.

High EZH2 expression has been described in various cancer types [6, 7], but only a few studies have investigated EZH2 expression in malignant mesothelioma [4, 5, 15, 21]. High EZH2 expression was demonstrated in 44.4% of the cases of malignant mesothelioma in the current study; which is less than what was recorded in Shinozaki-Ushiku et al. [4], whose frequency of high EZH2 expression was 57%, but was similar to the percentage reported in Yoshimura et al. [15], whose malignant mesothelioma cases showed high EZH2 expression in 44.7%. However, concerning RMH cases, previous papers [4, 15] including our current study, showed that none of the RMH cases showed high EZH2 expression, instead all showed no or low EZH2 expression.

Concerning Survivn expression in the current study, positive Survivin expression was detected in 67.9% of malignant mesothelioma cases, but none of the RMH cases showed Survivin positivity. This was similar to the finding previously described in Meerang et al. [22], who demonstrated Survivin positivity in 67.7% of their cases, while none of their cases of RMH showed Survivin expression. On the other hand, our results were lower than those reported by Hmeljak et al. [23], who detected nuclear Survivin positivity in 100% of their tumor samples.

Any discrepancies among different papers in the frequency of expression of any of the markers utilized in the current paper may be due to different sample sizes, differences in staining techniques, source of antibodies used for analysis, and the quantification technique. In our current study, we used fully automated immunohistochemical staining and commercially available antibodies from reputable sources. In addition, evaluation of nuclear reactivity was independently confirmed by both pathologists (the authors) of this study.

A combination of any two of the markers utilized in this study showed higher sensitivity compared to the individual use of either marker, whereas all combinations had 100% specificity; positive Survivin/BAP1 loss combination showed 75.3% sensitivity. This was unlike the percentage reported by Kushitani et al. [16], where positive Survivin and/or BAP1 loss combination showed a different sensitivity of 89.8% but similar specificity of 100% – the difference is probably due to different sample size and different techniques.

The current study reported that high EZH2 and/or BAP1 loss had 85.2% sensitivity. This was lower than the sensitivity reported by Shinozaki-Ushiku et al. [4] to the same combination (90%) but with similar specificity (100%), this could be attributed to the fact that they worked on both biphasic as well as epithelioid mesothelioma. The cases of this study, however, were only epithelioid mesothelioma. On the other hand, the sensitivity reported by Yoshimura et al. [15] (73.7%) was lower than that reported in the current paper, probably due to their relatively smaller sample size.

In the present study, the highest sensitivity for a combination of two markers was among high EZH2 and/or positive Survivin which reached 92.6%, with 100% specificity and 95.5% accuracy. To the best of our knowledge, this is the first study to assess the combined use of EZH2 and Survivin in malignant mesothelioma and RMH cases.

There was an inverse association between high EZH2 expression and each of BAP1 loss and positive Survivin expression. This inverse association enabled the combinations with high EZH2 (namely high EZH2 and/or BAP1 loss and high EZH2 and/or positive Survivin) to offer increased sensitivity in the differential diagnosis between EM and RMH (sensitivity of 85.2 and 92.6% respectively). These inverse associations might suggest that mechanisms underlying high EZH2 expression, BAP1 loss, and positive Survivin in EM may be distinct and need further elucidation. On the other hand, there was a highly significant direct association between BAP1 loss and positive Survivin, which might explain why their combination showed the least sensitivity 75.3%.

Concerning clinicopathological parameters of EM, emerging data has recommended the preferential use of two-tier grading of EM, IMIG stage and the need for commenting on the histological growth pattern of EM [10, 11, 24]. In our cohort of EM samples, we did not find any statistically significant relationships between the studied markers (BAP1, EZH2 and Survivin) and clinicopathological parameters of EM (growth pattern, two-tier grading, presence of necrosis, degree of nuclear atypia and IMIG stage). Interestingly, the frequency of EZH2, Survivin and BAP1 expression was similar in both low grade and high grade mesotheliomas, as well as in various growth patterns indicating that the combination of these markers is equally or similarly sensitive among all these morphological variants.

It is worth noting that most of the established markers for diagnosing malignant mesothelioma express cytoplasmic staining with variable intensity which makes it sometimes challenging to discriminate from non-specific staining. Thus, another advantage of the immunohistochemical markers (BAP1, EZH2 and Survivin) used in the current study is that they are all nuclear markers. New diagnostic immunohistochemical markers and techniques are needed for cases where established IHC markers cannot provide a clear entity diagnosis, and to improve treatment of malignant mesothelioma.

A recent study has shown a promising role for the loss of nuclear 5-hydroxymethylcytosine (5-hmC) immunohistochemical expression in mesothelioma, but stated that the need for new immunohistochemical markers still exists [25]. Therefore, the current study has demonstrated that using these new markers for malignant mesothelioma could greatly improve the diagnostic accuracy [26, 27].

Though it was beyond the scope of the current study, yet we found it intriguing to review the literature to find out the frequency of BAP1 loss, Survivin and EZH2 positivity in other malignancies that might pose differential diagnostic challenges with EM. While BAP1 loss was very rare in breast carcinoma [28], pancreatic ductal adenocarcinoma [29], non-small cell lung cancer [30] and colorectal adenocarcinoma [31], EZH2 positivity, on the other hand, was detected in advanced metastatic non-small cell lung cancer [32], 50% of primary pancreatic adenocarcinoma [33], more than 73.7% of primary colorectal carcinoma [34] and 49.8% of primary breast cancer [35]. Survivin was expressed in the cytoplasm of tumor cells in 59.3% of breast cancer [36] and 76.9% of pancreatic adenocarcinoma [37]. On the other hand, non-small cell lung cancer and colorectal cancer express Survivin nuclear positivity in 48.3 and 63.2% of tumor cells respectively [38, 39]. Further studies comparing the expression of BAP1, Survivin and EZH2 in different metastatic tumors and EM should be conducted.

One limitation of the present study is that it included pleural epithelioid mesothelioma and reactive mesothelial hyperplasia, the former being the commonest type of malignant mesothelioma and the latter being its mimic, however, more studies should be conducted involving other mesothelioma subtypes, other benign mesothelial lesions as well as other tumors of the pleura and metastatic tumors that pose diagnostic challenges.

In conclusion, adding EZH2, Survivin and BAP1 to the diagnostic IHC panel for differentiating pleural EM and RMH would increase the diagnostic sensitivity. Furthermore, the current study highlighted the promising potential diagnostic role of Survivin, especially when combined with EZH2, in discriminating pleural EM from RMH.

## Data Availability

The original contributions presented in the study are included in the article/Supplementary Material, further inquiries can be directed to the corresponding author.
